# A 5-Year Epidemiological Study of Monomicrobial Enterococcal Bloodstream Infection in a Population-Based Cohort

**DOI:** 10.1093/ofid/ofaf506

**Published:** 2025-08-20

**Authors:** David R Bayless, Larry M Baddour, Brian D Lahr, Grace A Hagan, Jenny J Cao, Daniel C DeSimone

**Affiliations:** Department of Internal Medicine, Mayo Clinic College of Medicine and Science, Rochester, Minnesota, USA; Division of Public Health, Infectious Diseases, and Occupational Medicine, Department of Medicine, Mayo Clinic College of Medicine and Science, Rochester, Minnesota, USA; Department of Cardiovascular Medicine, Mayo Clinic College of Medicine and Science, Rochester, Minnesota, USA; Department of Quantitative Health Sciences, Mayo Clinic College of Medicine and Science, Rochester, Minnesota, USA; Department of Internal Medicine, Mayo Clinic College of Medicine and Science, Rochester, Minnesota, USA; Department of Internal Medicine, Mayo Clinic College of Medicine and Science, Rochester, Minnesota, USA; Division of Public Health, Infectious Diseases, and Occupational Medicine, Department of Medicine, Mayo Clinic College of Medicine and Science, Rochester, Minnesota, USA; Department of Cardiovascular Medicine, Mayo Clinic College of Medicine and Science, Rochester, Minnesota, USA

**Keywords:** bloodstream infection, enterococci, mortality, population based study, Rochester Epidemiology Project

## Abstract

**Background:**

The incidence, epidemiology, and clinical characteristics of enterococcal bloodstream infection (BSI) have not previously been studied on a population-based level in the United States.

**Methods:**

We utilized the Expanded Rochester Epidemiology Project medical records linkage system to conduct a contemporary 5-year, retrospective, population-based study of monomicrobial enterococcal (ME) BSI among adult residents of 8 counties in southeast Minnesota from January 1, 2018 to December 31, 2022.

**Results:**

A total of 109 cases of ME-BSI were identified. The overall age- and sex-adjusted incidence rate was 6.7 per 100 000 person-years (95% confidence interval, 5.4–7.9). Multivariable analysis revealed that male sex (*P* < .001) and older age (*P* < .001) were each independently associated with increased incidence, whereas no temporal association was demonstrated (year effect, *P* = .475). The median age of incident cases was 76.8 years (interquartile range, 65.0–83.3). Urinary tract source was the most common presumed source (42.2%), and *Enterococcus faecalis* was the predominant (83.5%) pathogen. Overall, 14.4% of patients met modified Duke criteria for definite infective endocarditis. Cumulative mortality was 28.4% at 12 weeks and 43.1% at 1 year.

**Conclusions:**

In this population-based study, ME-BSI was significantly associated with older age and male sex. There was a high rate of mortality at 12 weeks and 1 year, with ∼1 out of 7 cases complicated by definite infective endocarditis. These findings underscore the importance of ongoing investigation of this syndrome.

## Teaser

Monomicrobial enterococcal bloodstream infection was significantly associated with older age and male sex in this retrospective study of adults in a midwestern cohort, which represents the first population-based study of enterococcal bloodstream infection in the United States. Approximately one in 7 cases (14.4%) was complicated by definite infective endocarditis, and cumulative mortality rates were 28.4% at 12 weeks and 43.1% at 1 year.

Enterococci are Gram-positive, facultative anaerobic bacteria that have become increasingly common pathogens over the past several decades [1, 2]. Enterococci cause a broad range of infections in humans, particularly healthcare-associated infections, bloodstream infection (BSI), urinary tract infections, hepatobiliary and other intra-abdominal infections, and infective endocarditis (IE) [1–3]. The majority of enterococcal infections are caused by *Enterococcus faecalis* and, to a lesser extent, *E. faecium*, the latter of which is the prototypical vancomycin-resistant *Enterococcus* (VRE) [4].

Enterococcal BSI most commonly occurs in older patients with multiple medical comorbidities and is associated with high mortality rates on longitudinal follow-up, with reported 30-day mortality rates often in the range of 10%–38% and 1-year mortality rates up to 40.2% [5–9]. Epidemiological studies outside the United States have described a rising incidence of enterococcal BSI in recent years, in addition to an increased incidence of BSI due to VRE [10–15]. Contemporary epidemiological data on the incidence of enterococcal BSI in the United States are more limited and population-level studies evaluating the incidence of enterococcal BSI have not, to our knowledge, been published from the United States [16]. Moreover, pertinent investigations in the United States and elsewhere have largely been conducted at tertiary care centers, which introduce a risk of referral bias that could have influenced study findings [17].

To better define the clinical profile of patients with monomicrobial enterococcal BSI (ME-BSI) in United States, we utilized the Expanded Rochester Epidemiology Project (E-REP) database to conduct a 5-year retrospective population-based study of adult patients in southeast Minnesota who developed ME-BSI.

The primary study outcome was the age- and sex-adjusted incidence rate of ME-BSI in a southeast Minnesota adult cohort. Secondary outcomes included the rates of complicating IE in ME-BSI and all-cause mortality at 12 weeks and 1 year. Cutoffs of 12 weeks and 1 year were selected to suitably represent shorter- and longer-term outcomes, respectively, in the context of a 5-year study duration.

## METHODS

Our study utilized the electronic E-REP system to determine the incidence of ME-BSI in southeast Minnesota. The REP is a medical records linkage system that maintains updated medical records for residents of Olmsted County, Minnesota, and affords the opportunity to conduct population-based epidemiological studies [18]. The REP was expanded in 2010 and now encompasses a total of 27 counties in southeast MN and Wisconsin (E-REP). The E-REP population is more diverse than the REP population and differs in persons living below the poverty line, non-White race, college education, and rural versus urban designation [19].

For incidence analyses, our study included only E-REP counties in which more than 90% of the county's census population had at least 1 visit available in a medical record from a health care institution participating in the E-REP. Eight counties in southeast MN (Olmsted, Dodge, Mower, Goodhue, Wabasha, Freeborn, Steele, and Waseca) were included, representing an annual population of ∼288 000 adults. Age- and sex-specific census figures derived from the E-REP data formed the basis of the incidence calculations.

Adults (age ≥18 years) diagnosed with an initial episode of ME-BSI between January 1, 2018 and December 31, 2022, were included if they had at least 1 blood culture positive for *Enterococcus* sp. from a standard peripheral venous blood culture collection. In addition, patients were included if they also had only 1 positive set of blood cultures with coagulase-negative staphylococci, *Corynebacterium* species, or *Cutibacterium acnes*. If a patient developed a recurrent or subsequent episode of ME-BSI during the study period, only the initial episode of ME-BSI was included among analyzed cases.

Exclusion criteria included patient denial of research authorization for medical records access; polymicrobial BSI with microorganisms in addition to enterococci (with the exceptions noted above) or with more than 1 *Enterococcus* species within 7 days of the primary ME-BSI; a previous episode of enterococcal BSI before the study period; and not residing within 1 of the 8 E-REP counties for at least 12 months prior to ME-BSI.

ICD-10 codes A41.81 (sepsis due to *Enterococcus*) and B95.2 (*Enterococcus* as the cause of diseases classified elsewhere) were used to identify patients in the study population with potential ME-BSI. Potential cases were individually reviewed in the Mayo Clinic electronic medical record (Epic Systems; Verona, WI) if sufficient medical records were available, as well as in the E-REP system (if needed) to confirm a first-time diagnosis of ME-BSI and to assess exclusion criteria. The E-REP system, which contains information regarding patient county of residence over time as well as clinical care received at Olmsted Medical Center, was utilized for medical records review when there was insufficient clinical information available in the Mayo Clinic electronic medical record, and to confirm that patients with ME-BSI had resided in 1 of the 8 E-REP counties in the study for at least 12 months prior to ME-BSI. Patients with ME-BSI who met inclusion criteria underwent medical record review, and data regarding host characteristics, microbiology, treatment plan, and clinical outcomes were collected. As enterococcal IE has previously been associated with colorectal neoplasms, data on colorectal neoplasm history among those diagnosed with ME-BSI were additionally collected [6, 20]. Data obtained from patient medical records were stored securely online using the Research Electronic Data Capture (REDCap) application (Vanderbilt University).

Cases of possible and definite IE were identified using the modified Duke Criteria [21]. Patients were defined as having ME-BSI relapse if they had a recurrence of BSI with the same species of *Enterococcus* within 12 weeks of the initial episode of ME-BSI, after clearance of initial blood cultures. The presumed source of infection was determined by the presence of either signs or symptoms of localizing infectious pathology, clinical suspicion of the treating team(s) as documented in available medical records, or by the presence of a positive bacterial culture with *Enterococcus* species from a usually sterile site with an identical species identification to that in the blood cultures [5]. Where there was significant ambiguity or an unrevealing workup, the source of infection was listed as unknown. The initial antibiotic regimen was defined as the antibiotic(s) used to treat ME-BSI and/or IE once sufficient microbiological information was available to tailor antibiotic therapy to the *Enterococcus* sp. The antibiotic course was recorded as not completed if treatment was interrupted (eg, due to death or drug toxicity) or if the regimen was changed due to concern for treatment failure. Antibiotic susceptibility testing of enterococcal BSI isolates was done in accordance with Clinical and Laboratory Standards Institute (CLSI) reference methods, and either CLSI or Food and Drug Administration minimum inhibitory concentration breakpoints were used to determine susceptibility.

Nondental invasive procedures performed in the 3 months prior to ME-BSI were recorded. Invasive procedures ranging from minor procedures to major surgeries were included. Due to the frequency of indwelling urinary catheter use in the hospital, initial urethral urinary catheter placement was not counted as a procedure; however, removal of an indwelling urinary catheter or exchange of an indwelling urinary catheter were counted as procedures if it was felt, based on review of medical records, that they were a potential source of bloodstream infection.

Nosocomial, health care-associated, and community-acquired BSI were defined according to the definitions provided by Friedman et al. [22]. Health care-associated BSI was defined as BSI diagnosed at hospital admission or within 48 hours of admission, with the patient meeting any of the following criteria: (1) received intravenous therapy at home, wound care or specialized nursing care, or had self-administered intravenous medical therapy in the 30 days before BSI; (2) attended a hospital or hemodialysis clinic or received intravenous chemotherapy in the previous 30 days; (3) hospitalized in an acute care hospital for 2 or more days in the previous 90 days; or (4) resided in a nursing home or long-term care facility [22]. Community-acquired BSI was defined as BSI diagnosed at hospital admission or within 48 hours of admission, but not meeting criteria for health care-associated BSI [22]. Nosocomial BSI was defined as BSI diagnosed more than 48 hours after hospital admission [22].

Mortality was attributable to ME-BSI if, on reviewing medical records, it was felt that ME-BSI had played a significant role in a patient's death. Attributable mortality was documented for patients deceased by 12 weeks after ME-BSI to better appreciate the short-term impact of ME-BSI on mortality.

### Patient Consent Statement

The study was exempt from patient consent as it did not involve patient contact or methods requiring consent. The study was approved by the Mayo Clinic Institutional Review Board (IRB# 23-008134).

### Statistical Analysis

Descriptive statistics on baseline patient characteristics are reported as median (IQR [interquartile range]) for continuous variables and percentage (number) of patients for categorical variables. For clinical outcomes of interest, cumulative event rates were estimated using the cumulative incidence function estimator for outcomes subject to competing risks (ie, hospital discharge and 12-week relapse, for which death is a competing risk) and using the Kaplan–Meier (inverse survival) estimator for death up to 1 year from ME-BSI diagnosis.

Incidence rates of ME-BSI were calculated by dividing the number of cases by the population person-years per stratum, expressed per 100 000 person-years. Strata were formed by sex and single-year-of-age (from 18 to 100) so that the overall rates could be standardized to the age and sex distribution of the 2020 US population. The 95% confidence intervals (CIs) were calculated assuming that the count of incident cases follows a Poisson distribution. To explore univariable (unadjusted) relationships with incidence, we repeated the incidence calculations stratified by sex, age group (18–49, 50–59, 60–69, 70–79, ≥80 years old), year (2018, 2019, 2020, 2021, and 2022), season (winter, spring, summer, and fall), and geographic region (Olmsted County, all other counties).

For further examination, variables were entered into a Poisson regression model to determine which factors independently influenced ME-BSI incidence. The dependent variable was the number of cases, and the independent variables were age, sex, calendar year, and geographic region, with the log count of person-years included as an offset variable. An input data set suitable for Poisson modeling was obtained by stratifying the data into subsets according to the levels of these independent variables, with age and calendar time each divided into yearly intervals so that these effects could be analyzed on a continuous scale. Our analysis strategy prespecified some modeling flexibility that allowed certain assumptions to be relaxed. Namely, nonlinear terms were fitted for both continuous variables (using a restricted cubic spline function for age and a quadratic effect of year), and a linear interaction was allowed between age and sex. From the model, the partial tests for association of each predictor with incidence were reported, and incidence rate ratios (IRR) were derived from the regression coefficients to provide adjusted measures of effect. A second multivariable model assuming linearity and additivity of effects was used to estimate an average effect of age per decade increase adjusted for the other factors. All analyses were performed using the statistical programming language R version 4.2.2 (R Foundation, Vienna, Austria).

## RESULTS

A total of 203 cases of *Enterococcus* BSI were detected within a preliminary cohort of 1246 patients identified by the E-REP system based on ICD codes A41.81 and B95.2 (Supplementary Figure 1). Overall, 109 cases of incident ME-BSI meeting study inclusion criteria were identified from the 203 BSI cases and included in the final study cohort (Supplementary Figure 1). 105 patients had ME-BSI in the strict sense, whereas 4 patients had 1 positive set of blood cultures with coagulase-negative staphylococci, *Corynebacterium* species, or *Cutibacterium acnes* coincident with enterococcal BSI. 94 cases of enterococcal BSI were excluded due to not meeting inclusion criteria or meeting exclusion criteria. For 1 patient with reported *E. faecalis* BSI, research authorization for medical records access was partially denied so the patient was excluded.

The median age and Charlson Comorbidity Index of patients within the study cohort were 76.8 years (IQR, 65.0–83.3) and 6 (IQR, 4–8), respectively (Table 1). Overall, 63.3% of patients were male and 93.5% were White (Table 1). Most (71.6%) ME-BSI cases were healthcare-associated (Table 1). Common comorbid conditions in the cohort included smoking history (54.1%), obesity (46.8%), diabetes mellitus (45.9%), and valvular heart disease (41.6%).

**Table 1.** Clinical Characteristics of Patients Within 8 Counties in the E-REP System With Incident Monomicrobial Enterococcal Bloodstream Infection Between 2018 and 2022

Characteristic	*N*	Overall
Age at ME-BSI, y	109	76.8 (65.0–83.3)
Male sex	109	63.3% (69)
Race	108	…
White	…	93.5% (101)
Black	…	1.9% (2)
Asian	…	1.9% (2)
Native Hawaiian or Other Pacific Islander	…	0.9% (1)
Other	…	1.9% (2)
Hispanic ethnicity	107	1.9% (2)
Obesity (BMI >30 kg/m^2^) at time of ME-BSI	109	46.8% (51)
Smoking history	109	54.1% (59)
History of IE	109	2.8% (3)
Chronic kidney disease with eGFR < 30	109	11.9% (13)
On maintenance dialysis	109	5.5% (6)
Injection drug use	109	0.9% (1)
Immunocompromising condition^a^	109	22.9% (25)
Immunocompromising medications^b^	109	21.1% (23)
Inflammatory bowel disease	109	4.6% (5)
Congestive heart failure	109	33.9% (37)
Chronic obstructive pulmonary disease	109	15.6% (17)
Valvular heart disease^c^	101	41.6% (42)
Congenital heart disease	109	5.5% (6)
Cirrhosis	109	5.5% (6)
Diabetes mellitus	109	45.9% (50)
Charlson comorbidity index	109	6 (4–8)
History of colorectal neoplasm^d^	109	38.5% (42)
Adenocarcinoma^e^	42	16.7% (7)
Tubular adenoma	42	73.8% (31)
Adenoma with villous features or high-grade dysplasia	42	7.1% (3)
Nondysplastic sessile serrated polyp	42	19.0% (8)
Other or unspecified adenoma	42	4.8% (2)
Presence of cardiac implantable electronic device^f^	109	19.3% (21)
Presence of vascular graft/stent	109	25.7% (28)
Presence of an indwelling intravenous catheter^g^	109	15.6% (17)
Nondental invasive procedure within 3 m prior to ME-BSI	109	56.9% (62)
History of valvular procedure prior to ME-BSI	109	20.2% (22)
Type of procedure	22	…
TAVR	…	18.2% (4)
Open aortic valve replacement	…	36.4% (8)
Open mitral valve replacement	…	13.6% (3)
Other	…	31.8% (7)
Prosthetic valve in place	109	18.3% (20)
Type of prosthetic: bioprosthetic	20	60.0% (12)
Echocardiography obtained at time of ME-BSI diagnosis	109	48.6% (53)
Type of echocardiography	53	…
Transthoracic echocardiogram	…	43.4% (23)
Transesophageal echocardiogram	…	30.2% (16)
Both	…	26.4% (14)
ME-BSI classification	109	…
Healthcare-associated	…	71.6% (78)
Community-acquired	…	21.1% (23)
Nosocomial	…	7.3% (8)

Past medical conditions refer to those present at the time or before documentation of bloodstream infection.

Data are presented as median (25th percentile–75th percentile) for continuous variables and percentage (number) for discrete variables. *N* represents the number of patients with available information.

^a^Includes advanced-stage solid cancer, hematologic malignancy, history of bone marrow transplant, and HIV infection.

^b^Includes chronic glucocorticoids (any dose), transplant rejection agents, chemotherapy, and disease-modifying antirheumatic agents (DMARDs).

^c^Indicates a known history of more than mild valvular stenosis or regurgitation.

^d^Denotes the neoplasm(s) diagnosed on most recent screening or targeted diagnostic study before ME-BSI.

^e^Included appendiceal carcinoma in 1 case.

^f^Includes pacemakers and automatic implantable cardioverter defibrillators.

^g^Includes chemotherapy infusion ports, peripherally inserted central catheters (PICC lines), and hemodialysis catheters (tunneled and nontunneled).

More than half (56.9%) of the patients had undergone a nondental invasive procedure within 3 months prior to BSI onset (Table 1). The most common procedures were urologic procedures (including radical cystectomy, cystoscopy, ureteroscopy, lithotripsy, nephrostomy tube placement, and indwelling urinary catheter removal or exchange) in 21 cases (33.9% of total procedures within 3 months prior to BSI) and endoscopic retrograde cholangiopancreatography (ERCP) in 9 cases (14.5% of total procedures). In 14 cases (22.6% of total procedures), patients had undergone nonurologic major surgery in the 3 months prior to BSI, including open-heart valvular replacement, exploratory laparotomy, or hip fracture repair.

The 3 most common sources of BSI were urinary tract pathology (42.2%), unknown (24.8%), and intra-abdominal pathology (22.9%) (Table 2). *E. faecalis* was the predominant pathogen (83.5% of cases). Among the enterococcal isolates identified, 8 (47.1%) of 17 *E. faecium* isolates demonstrated vancomycin resistance and 12 (70.6%) of 17 *E. faecium* isolates demonstrated high-level penicillin or ampicillin resistance. There were no *E. faecalis* isolates with either vancomycin or high-level penicillin or ampicillin resistance.

**Table 2.** Microbiological and Treatment Data for Incident Monomicrobial Enterococcal Bloodstream Cases in the E-REP Study Population From 2018 to 2022

Characteristic	*N*	Overall
Infection source	109	…
Urinary tract	…	42.2% (46)
Skin and soft tissue	…	1.8% (2)
Intra-abdominal (nonurinary)	…	22.9% (25)
Pneumonia	…	0.9% (1)
Central nervous system	…	0.9% (1)
Infectious source unknown	…	24.8% (27)
Central catheter-associated	…	5.5% (6)
Prosthetic/hardware associated	…	0.9% (1)
Blood culture results	109	…
*E. faecalis*	…	83.5% (91)
*E. faecium*	…	15.6% (17)
*E. avium*	…	0.9% (1)
Vancomycin resistance	107	7.5% (8)
*E. faecium*	…	100% (8)
High-level penicillin or ampicillin resistance	108	11.1% (12)
*E. faecium*	…	100% (12)
High-level aminoglycoside resistance	102	14.7% (15)
*E. faecalis*	…	86.7% (13)
*E. faecium*	…	13.3% (2)
Time to ME-BSI clearance, d	98	2 (1–3)
Initial antibiotic regimen^a^	104	…
IV vancomycin	…	25.0% (26)
IV ampicillin plus ceftriaxone	…	24.0% (25)
IV ampicillin	…	21.2% (22)
IV piperacillin–tazobactam	…	11.5% (12)
IV daptomycin	…	6.7% (7)
PO or IV linezolid	…	3.8% (4)
IV ampicillin–sulbactam	…	3.8% (4)
Other	…	3.8% (4)
Completed full course of therapy	102	79.4% (81)
Full course not completed	21	…
Death	…	52.4% (11)
Patient unable or unwilling to complete antibiotic course^b^	…	28.6% (6)
Treatment failure	…	9.5% (2)
Nephrotoxicity	…	4.8% (1)
Drug hypersensitivity reaction	…	4.8% (1)
Duration of antibiotics received, wk	102	2 (2–3)
Completed antibiotics in outpatient setting	81	79.0% (64)

Abbreviations: PO, by mouth; IV, intravenous.

Data are presented as median (25th percentile–75th percentile) for continuous variables and percentage (number) for discrete variables. *N* represents the number of patients with available information.

^a^An antibiotic regimen was not defined for 5 patients. For 2 of these cases, positive enterococcal blood culture was treated as a contaminant; for 2, antibiotic regimen was not defined due to patient death; in the final case, the antibiotic regimen was not clearly targeted toward *Enterococcus* sp.

^b^Includes patients who either elected to transition to hospice/comfort cares, or whose family decided this on their behalf.

Overall, 24% of BSI cases were treated with the combination of ampicillin and ceftriaxone (Table 2). Specifically, 14 cases of definite IE were treated with ampicillin and ceftriaxone; 10 cases of possible IE were treated with ampicillin and ceftriaxone; and 1 patient not meeting modified Duke criteria for IE received ampicillin and ceftriaxone, due to initial clinical concern for IE. Only 1 patient with definite IE was not treated with ampicillin and ceftriaxone; in this case, the regimen consisted of ampicillin, gentamicin, and daptomycin for *E. faecium* IE of the mitral valve, with a valvular annuloplasty band in place. Median duration of antibiotic therapy was 2 weeks (IQR, 2–3), although cases of suspected or confirmed enterococcal IE were most commonly treated with 4–6 weeks of antibiotic therapy. Among patients with available data (*n* = 102), 11 patients (10.8%) received antibiotics for 3–5 weeks, 16 patients (15.7%) received antibiotics for 6 weeks, and 3 patients (2.9%) received antibiotics for greater than 6 weeks. Among patients alive at 12 weeks (*n* = 78, with data missing for 4), 7 (9.5%) received antibiotics for 3–5 weeks, 14 (18.9%) received antibiotics for 6 weeks, and 3 (4.1%) received antibiotics for >6 weeks.

**Figure 1.**
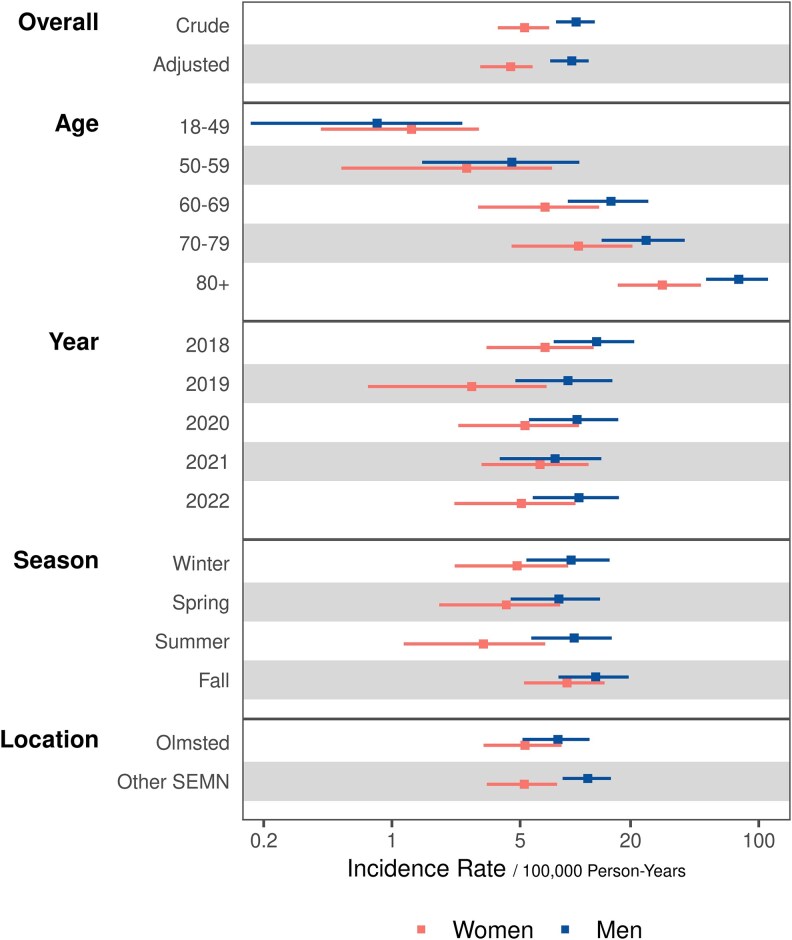
A graph showing the incidence of monomicrobial enterococcal bloodstream infection in the study population when stratified by sex and the following factors: age, study year, season of the year, and geographic location. The top panel shows crude and age-adjusted incidence rates (per 100 000 person-years) by sex, demonstrating significantly higher incidence among men. Other panels show crude sex-specific incidence rates when stratified separately by other factors. Patterns can be assessed by examining the uniformity of rates across strata of a given factor. These rates varied markedly across age groups, with an increasing incidence rate with age, but otherwise showed a lack of temporal, seasonal, or geographic variation. Center blocks represent point estimates and bars represent 95% confidence limits of incidence rates. SEMN, southeast Minnesota.

The overall age- and sex-adjusted incidence rate of ME-BSI cases was 6.7 per 100 000 person-years (95% CI 5.4–7.9). Supplementary Table 1 presents the incidence rates stratified by sex and age groups. Men had a higher age-adjusted incidence rate, 9.6 (95% CI 7.3–11.8) cases per 100 000 person-years, compared with women, 4.4 (95% CI 3.0–5.8; Supplementary Table 1). The incidence of ME-BSI overall and in both sexes increased with older age, rising each step in age group and peaking among persons 80 years or older (Supplementary Table 1; Figure 1). When stratifying incidence separately by other descriptors, no discernible patterns of temporal, seasonal, or geographic variation were observed (Figure 1).

**Figure 2.**
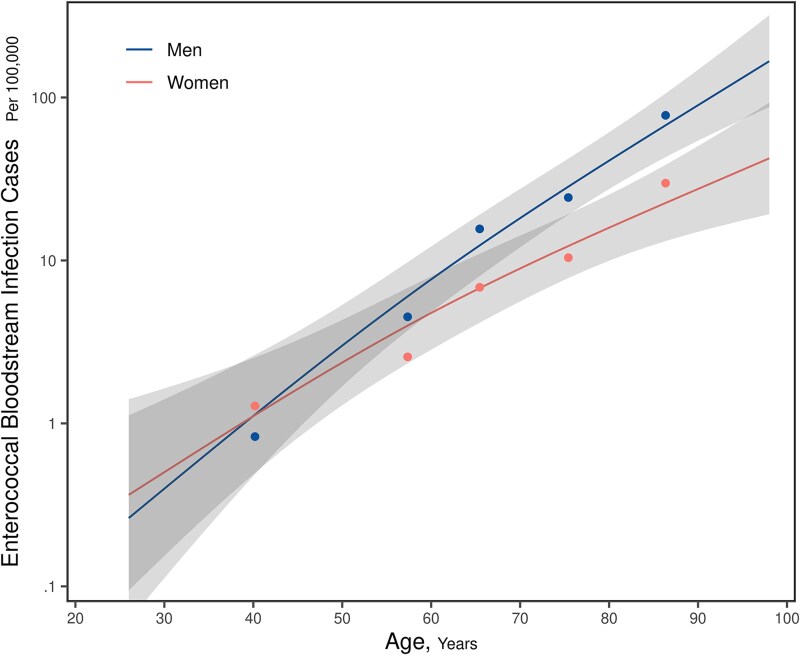
A graph showing the predicted incidence of monomicrobial enterococcal bloodstream infection in the study population when stratified by sex and according to age. The graph shows rising incidence rates with age for both sexes. The fitted curves (and 95% confidence limits) were derived from a multivariable Poisson regression model with the other independent variables fixed at representative values (geographic region: Olmsted County; year: 2020). Age was modeled flexibly with a restricted cubic spline to allow for nonlinear relationships with the response (log-rate) and a linear interaction with sex. The curves in this plot illustrate the exponentially increasing incidence rates with age for both sexes. The nonparallel slopes of these curves indicate a possible interaction, and the age-related rise in incidence appears to be steeper in men than in women; however, the age-by-sex interaction effect was not statistically significant (*P* = .086). Dots represent the crude incidence rates computed for age groups (18–49, 50–59, 60–69, 70–79, and ≥80 y), which are provided here as a crude verification of the model fit. The significant increase in ME-BSI incidence with age highlights the importance of infection prevention and early detection of infection in older patients.

Multivariable analysis using Poisson regression showed that older age and male sex were each independently associated with increased incidence of ME-BSI (both *P* < .001). Age-related increase in incidence was more pronounced in men than in women, although the age-by-sex interaction effect was not statistically significant (*P* = .086; Figure 2). On average, the risk of advancing age was associated with a 2.2-fold rise (95% CI 1.9–2.4) in ME-BSI incidence with each decade of life. Male sex was also associated with a more than 2-fold higher risk of incident ME-BSI (IRR: 2.3 [95% CI 1.6–3.5]) after adjusting for the other covariates. Neither the calendar year (*P* = .475) nor the geographic region (*P* = .923) variables contributed significantly to ME-BSI incidence.

Overall, 44.2% of incident ME-BSI patients with available data (46/104) met criteria for possible or definite IE, of whom 32.6% (14.4% of the total cohort) had definite IE (Table 3). Among the 4 patients with a history of transcatheter aortic valve replacement (TAVR), 1 met criteria for definite IE and 2 met criteria for possible IE. Over the 12 weeks following ME-BSI diagnosis, 6 patients developed ME-BSI relapse and 31 patients died (2 after relapse), corresponding to 12-week relapse and mortality rates of 5.6% and 28.4%, respectively (Table 3; Supplementary Figure 2). Seventy-three patients completed 12-week follow-up without relapse (1 patient had incomplete follow-up data and was censored), for a relapse-free survival rate of 67.6% (Supplementary Figure 2). A total of 47 patients died during the 1-year follow-up period, corresponding to a cumulative all-cause mortality rate of 43.1% (Table 3; Supplementary Figure 2). Among patients deceased by 12 weeks after ME-BSI, mortality was attributable to ME-BSI in 35.5% of cases (11 of 31 patients).

**Table 3.** Clinical Outcomes of Patients With Monomicrobial Enterococcal Bloodstream Infection in the Study Population

Outcome	*N*	Overall
Cardiac complications	98	11.2% (11)
Type of complication^a^	11	…
Abscess formation	…	9.1% (1)
Valvular insufficiency	…	63.6% (7)
Cusp perforation	…	9.1% (1)
Two or more of the above	…	18.2% (2)
Other complications^b^	98	7.1% (7)
Septic emboli	99	9.1% (9)
Septic emboli location	9	…
Brain	…	77.8% (7)
Spleen	…	11.1% (1)
Lungs	…	11.1% (1)
Infective endocarditis	104	44.2% (46)
Certainty of IE: definite	46	32.6% (15)
Postdiagnosis hospital LOS, d	108	7 (5–14)*
Relapse, wk = 12	109	5.6% (6)*
Cumulative mortality	109	…
In-hospital	…	8.3% (9)
12 wk	…	28.4% (31)†
1 y	…	43.1% (47)†
Attributable mortality (if deceased at 12 wk after ME-BSI)	31	35.5% (11)

LOS, length of stay.

Values represent simple percentages for binary outcomes and cumulative incidence % (cumulative number of events) for time-to-event outcomes. Time-to-event estimators used: *Cumulative incidence function event rate or quartile estimates for outcomes subject to competing risks; †Inversed Kaplan–Meier survival estimates for time-specific mortality. *N* is the number of patients with available information.

^a^In 1 case, there was severe valvular stenosis in addition to valvular insufficiency. In a different case, the patient had both heart failure and severe valvular insufficiency as complications.

^b^Examples of other complications include local (as opposed to metastatic) liver and kidney abscesses as well as spinal infection.

## DISCUSSION

To our knowledge, this is the first population-based epidemiologic study of ME-BSI conducted in the United States. Between 2018 and 2022, there were 109 incident cases of ME-BSI in this large, geographically defined population in southeastern Minnesota. The age- and sex-adjusted incidence rate of ME-BSI was 6.7 per 100 000 person-years. Older age and male sex were independently associated with increased incidence of ME-BSI, and there was no significant temporal trend during the 5-year study period. The percentage of incident ME-BSI cases with definite IE was 14.4%. The 12-week and 1-year all-cause mortality rates were alarmingly high at 28.4% and 43.1%, respectively, with 35.5% of deaths occurring by 12 weeks after ME-BSI attributable to the infection.

Population-based studies of enterococcal BSI incidence outside the United States conducted within the past 25 years have reported comparable estimates to that of the current investigation, including an incidence rate of 6.6/100 000 person-years for only monomicrobial *E. faecalis* BSI in British Columbia, 19.6/100 000 person-years in Denmark for monomicrobial and polymicrobial BSI due to *E. faecalis* and *E. faecium*, 19.9/100 000 person-years for enterococcal BSI in the Barwon region of Australia, and 6.9/100 000 for monomicrobial and polymicrobial enterococcal BSI in Calgary [5, 6, 17, 23]. Whereas studies outside the United States have reported an increasing incidence of enterococcal BSI in recent years, our investigation did not find a significant change in ME-BSI incidence rates. Reasons for this difference may reflect demographic differences in study populations, either on a national level (ie, pertaining to the US population in general) or a more regional level (ie, pertaining to the southeast MN population). Moreover, our study was limited to only 5 years and thus was not designed to detect gradual but potentially important changes in incidence that a longer period of study might reveal. As the majority of population-based research on enterococcal BSI has been conducted in populations or centers outside of the United States, additional population-based research in the United States would allow comparison with our own data (representative of a particular region of the midwest US), as well as further comparison with data available from outside the United States.

ME-BSI was more common in males than females in our cohort, a similar trend to what has been reported in previous epidemiological studies [5, 17, 23]. Specific mechanisms underlying the higher prevalence of enterococcal BSI in men compared to women have not been studied, but based on more general data regarding sex differences in bacterial and BSI risk, higher rates of enterococcal BSI could relate to biological differences in susceptibility to BSI, higher rates of cardiovascular and other comorbid conditions in men, and behavioral differences such as current or prior cigarette smoking, among other factors [24, 25]. Also consistent with previous studies is the rising incidence rate of enterococcal BSI with age [17, 23]. This is most likely multifactorial due to increasing comorbidities and susceptibility to infection with age as well as greater healthcare exposure. A strong association between enterococcal BSI and healthcare exposure is suggested by our finding that the majority (78.9%) of ME-BSI cases were either healthcare-associated or nosocomial, with similar rates reported in the population-based studies of enterococcal BSI by both Billington et al. and Bright et al. (80% and 84.9%, respectively) [17, 23].

In relation to this, the most common suspected source of ME-BSI in our study was urinary tract (42.2%), with unknown source (24.8%) and nonurinary tract intra-abdominal (22.9%) also comprising a significant proportion of cases. These results are similar to findings from previous studies and suggest that assessment for urinary tract and intra-abdominal sources plays a critical role in evaluating enterococcal ME-BSI [5, 8, 17, 26]. Central venous catheter-associated infections were not common (5.5%) in our cohort. The multicenter VENOUS I study in the United States, which evaluated patients with positive blood cultures for *E. faecalis* or *E. faecium* in 11 hospitals, identified central line infection as the infection source in 22.16% of vancomycin-sensitive enterococcal BSIs and 30.36% of vancomycin-resistant enterococcal BSIs [9]. Rates of central venous catheter-associated BSIs could differ between our and other studies such as VENOUS I for multiple reasons, such as differences in rates of central venous catheter use (eg, owing to differences in patient populations), institutional variation in catheter management, and variability in data collection when only 1 source of BSI is recorded.

Regarding ME-BSI of unknown source, an important consideration is that more than one-third (38.5%) of patients with ME-BSI in our cohort had a previous diagnosis of colorectal neoplasm, and previous data have suggested a higher rate of colorectal neoplasms in patients with *E. faecalis* IE [20]. In the population-based study of enterococcal BSI by Cabiltes et al., in which 68.4% of patients were men and the median age was 71 years, 9 of 12 patients who were referred for colonoscopy after being diagnosed with enterococcal BSI were found to have previously undiagnosed colorectal neoplasia, though the significance of this result is difficult to interpret given small sample size and higher expected rates of colorectal neoplasia in older patients [6]. It remains the case that patients with enterococcal BSI, in the opinion of some experts, should be considered for diagnostic colonoscopy to assess for a portal of entry. Colonoscopy should particularly be considered if an initial source of BSI is not identified, or if the patient has not undergone age-appropriate colorectal cancer screening.

The rate of definite IE with ME-BSI in our cohort was 14.4%, with nearly half (48.6%) of patients having undergone echocardiography, whether transthoracic, transesophageal, or both. Other studies have reported similar (15%–33%) rates of IE in enterococcal BSI [5, 6, 27–29], though some studies have found rates <10% [9, 30]. The study by Lai et al., based on a cohort of patients in Hong Kong, found an IE rate of 3.39% among 2535 episodes of *E. faecalis* BSI, but the authors noted a low rate of echocardiogram use (3.83%) [30]. Some of the above studies were comparative and demonstrated that risk of IE in the setting of enterococcal BSI was higher than that for other bacteria, such as *Staphylococcus aureus* and *Streptococcus* species. Among Danish cohorts, Østergaard et al. found an IE prevalence of 16.7% for *E. faecalis* BSI, with 10.1% IE prevalence for *S. aureus* and 7.3% for *Streptococcus* species; Andersen et al. found a prevalence of 33% for IE among those with *E. faecalis* BSI compared to 23% for nonbeta-hemolytic streptococci and 12% for *S. aureus* [28, 29]. Because the incidence of definite IE in our cohort could be underestimated due to use of screening echocardiography in less than half of ME-BSI cases, our results support maintaining a low threshold for obtaining at least transthoracic echocardiography to evaluate for complicating IE in patients with ME-BSI, particularly if IE risk-enhancers such as male sex, persistent bacteremia, presence of a prosthetic heart valve, or immunosuppression are present [27, 28]. The NOVA and DENOVA risk stratification scores have been clinically studied and may guide decision making regarding need for TTE and/or TEE in patients with enterococcal BSI [31].

It is worth noting that incidence rates of enterococcal IE have risen in recent decades, and enterococci are the third most common bacterial cause of IE [32, 33]. Moreover, enterococci have been the most common cause of IE following TAVR in multiple investigations, which has prompted some experts to advocate for antibiotics other than cefazolin as surgical site infection prophylaxis at the time of TAVR [34, 35].

In summary, the results of our study, taken in conjunction with those from the literature, suggest ME-BSI is a medically urgent condition associated with high longitudinal mortality rates. Clinical implications of our work regarding care for those with ME-BSI or at high risk of developing it include the importance of early identification and treatment of ME-BSI in higher risk patients, including older patients, males, and those with significant healthcare exposure or undergoing an invasive procedure; appropriate evaluation for potential sources of enterococcal BSI, with consideration of colonoscopy particularly in those without an identified source; and maintaining a low threshold to evaluate for complicating IE in patients with ME-BSI.

## LIMITATIONS

Our study has multiple limitations. Regarding demographics, applicability of our results to predominantly non-White populations may be reduced as our study had limited representation of non-White patients, with 93.5% of the cohort consisting of White patients. Additionally, the midwest United States location in which the study cohort was based does not host any major urban centers, which can be a source of reduced applicability to more urban patient populations.

Although medical records were available for more than 90% of residents of the 8 counties under study in the E-REP system, this does exclude adults in the region who had not received care at an institution participating in E-REP data collection. Adults who denied research authorization were also excluded, and 1 patient with reported *E. faecalis* BSI was found in the preliminary study cohort but partially denied research authorization and was excluded from the final study cohort. Moreover, we are unable to determine the number of patients who were not captured by the E-REP system for the initial ICD code search. These observations suggest there are an undetermined number of cases of ME-BSI within the study counties that we were unable to capture and analyze. More generally, the retrospective design of the study as well as reliance on medical documentation and diagnostic coding could have led to incomplete or inaccurate assessment of certain study variables.

Given our focus on population-level data and limited sample sizes in the study, we did not stratify our data by subpopulation characteristics, such as by comparing patient characteristics of those with possible or definite IE compared to those without IE, or by comparing patients with *E. faecalis* BSI to those with *E. faecium* BSI. Notably, previous studies have identified differences in the epidemiology and outcomes of *E. faecalis* BSI and *E. faecium* BSI [5, 17, 36, 37], with *E. faecium* BSI frequently associated with higher mortality rates than *E. faecalis* BSI [5, 17, 36].

The 5-year duration of the study limits the ability to detect changes in ME-BSI incidence and outcomes over a longer period. Similarly, limiting assessment of patient outcomes to 1-year after ME-BSI incidence precludes assessment of potentially important trends in longer-term outcomes of ME-BSI. We also note that the rate of complicating IE in our cohort could be underestimated as only 48.6% of patients underwent echocardiographic evaluation.

Finally, we opted to utilize the modified (2000) Duke criteria to define IE as the 2023 Duke-ISCVID criteria were available after initiation of our project. The 2023 criteria included a major revision that was also the focus of a 2022 prospective, multicenter investigation in regard to designating *E. faecalis* as a microorganism that commonly causes IE, in contrast to the 2000 modified Duke criteria, which limited the major criteria to include only “community-acquired enterococci, in the absence of a primary focus” [21, 38].

## CONCLUSIONS

In this population-based study, ME-BSI was significantly associated with male sex and older age. 14.4% of patients developed definite IE and 1-year all-cause mortality rate was 43.1%. The high mortality rates associated with ME-BSI underscore the importance of ongoing investigation of this syndrome, with attention to incidence rates, prevention, early recognition and management.

## Supplementary Material

ofaf506_Supplementary_Data

## Supplementary Data

Supplementary materials are available at *Open Forum Infectious Diseases* online. Consisting of data provided by the authors to benefit the reader, the posted materials are not copyedited and are the sole responsibility of the authors, so questions or comments should be addressed to the corresponding author.
