# Supplementary material for: A 5-Year Epidemiological Study of Monomicrobial Enterococcal Bloodstream Infection in a Population-Based Cohort
Source: Open Forum Infect Dis. 2025 Aug 20;12(9):ofaf506. doi: 10.1093/ofid/ofaf506 (PMC12415331; doi:10.1093/ofid/ofaf506)
Supplement: ofaf506_Supplementary_Data [file ofaf506_supplementary_data.docx]

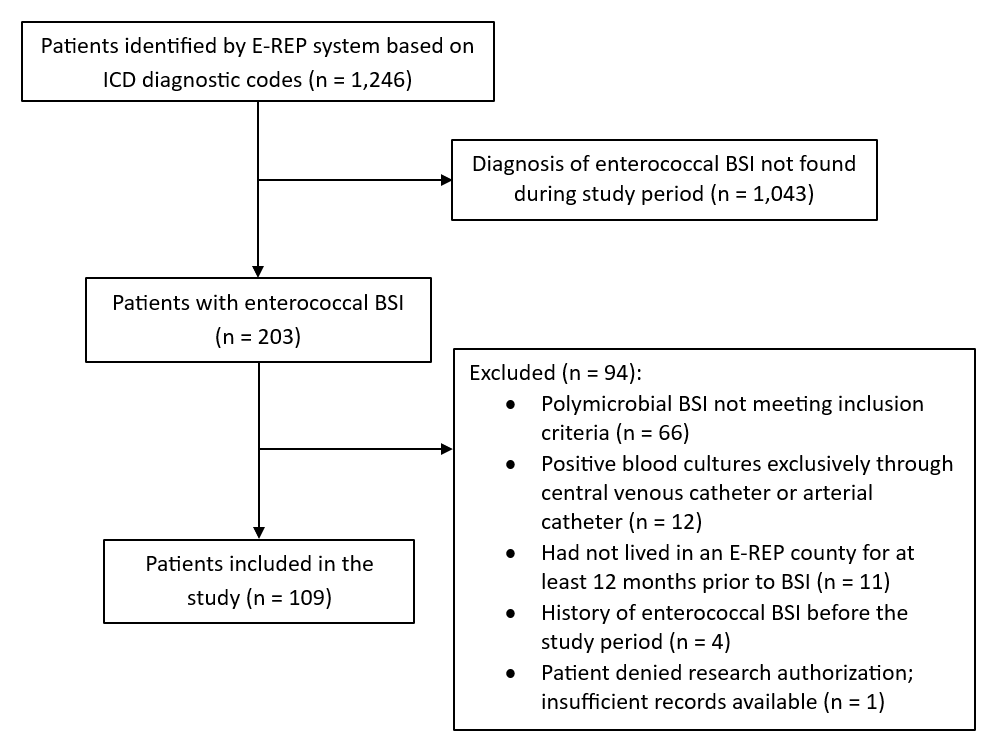


**Supplementary Figure 1**: Flow chart demonstrating number of patients screened for study inclusion, number of cases excluded with reasons for exclusion, and final number of patients included in the study. Patient records were invidually reviewed to determine study inclusion. BSI = bloodstream infection. E-REP = Expanded Rochester Epidemiology Project.


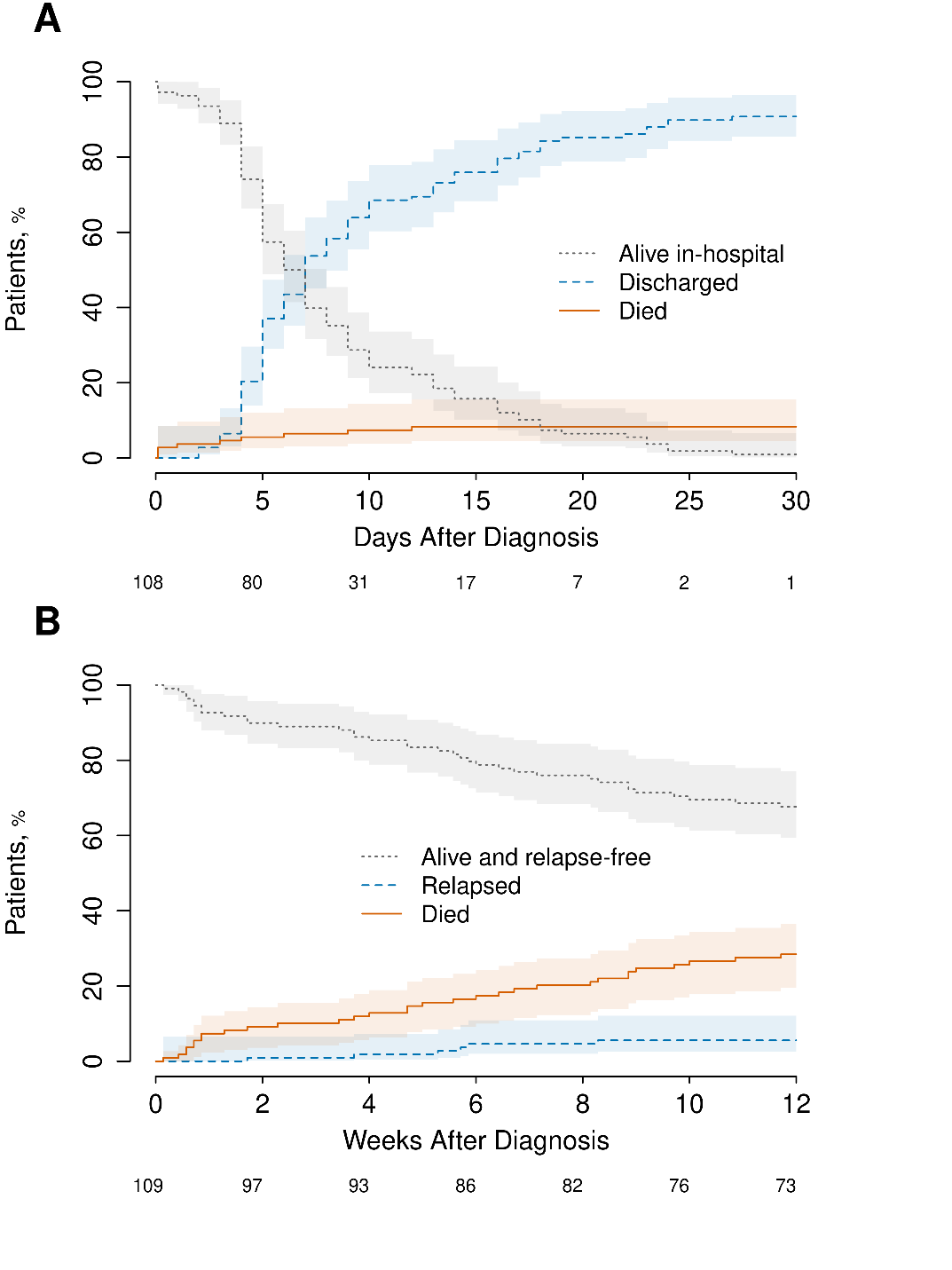


**Supplementary Figure 2**: Cumulative incidence of (A) hospital discharge and (B) 12-week ME-BSI relapse. Blue dashed lines represent the primary event of interest in the respective panels; the discharged and relapsed curves are cumulative incidence curves of hospital discharge and relapse, respectively, each accounting for the competing risk of death. The death curve is a (in-hospital or 12-week) cumulative mortality curve based on inversed Kaplan-Meier survival estimates. The shaded bands surrounding the dashed and solid lines correspond to 95% confidence limits.

**Supplementary Table 1:** incidence rates of monomicrobial enterococcal bloodstream infection in the study population (N = 109)

|  |  |  | **Female** | |  |  | **Male** | |  |  | **Total** | |
| --- | --- | --- | --- | --- | --- | --- | --- | --- | --- | --- | --- | --- |
| **Age Group** |  |  | **No.** | **IR** |  |  | **No.** | **IR** |  |  | **No.** | **IR** |
| 18-49 |  |  | 5 | 1.3 |  |  | 3 | 0.8 |  |  | 8 | 1.1 |
| 50-59 |  |  | 3 | 2.6 |  |  | 5 | 4.5 |  |  | 8 | 3.5 |
| 60-69 |  |  | 8 | 6.8 |  |  | 17 | 15.6 |  |  | 25 | 11.1 |
| 70-79 |  |  | 8 | 10.4 |  |  | 16 | 24.3 |  |  | 24 | 16.8 |
| 80-100 |  |  | 16 | 29.8 |  |  | 28 | 77.7 |  |  | 44 | 49.0 |
| Overall |  |  | 40 | 4.4 (3.0-5.8)^1^ |  |  | 69 | 9.6 (7.3-11.8)^1^ |  |  | 109 | 6.7 (5.4-7.9)^2^ |
| No., number of incident ME-BSI cases; IR, incidence rate (number of incident cases per 100,000 person-years) ^1^ Age-adjusted incidence rate (95% confidence interval) ^2^ Age- and sex-adjusted incidence rate (95% confidence interval) | | | | | | | | | | | | |
